# The clinically relevant CYP2C8*3 and CYP2C9*2 haplotype is inherited from Neandertals

**DOI:** 10.1038/s41397-022-00284-6

**Published:** 2022-07-02

**Authors:** Sigrid Haeggström, Magnus Ingelman-Sundberg, Svante Pääbo, Hugo Zeberg

**Affiliations:** 1 Department of Neuroscience, Karolinska Institutet, SE-17177 Stockholm, Sweden; 2 Department of Physiology and Pharmacology, Karolinska Institutet, SE-17177 Stockholm, Sweden; 3 Department of Evolutionary Genetics, Max Planck Institute for Evolutionary Anthropology, D-04103 Leipzig, Germany; 4 Human Evolutionary Genomics Unit, Okinawa Institute of Science and Technology, Okinawa, 904-0495 Japan

**Keywords:** Drug regulation, Risk factors

## Abstract

Genetic variation in genes encoding cytochrome P450 enzymes influences the metabolism of drugs and endogenous compounds. The locus containing the cytochrome genes *CYP2C8* and *CYP2C9* on chromosome 10 exhibits linkage disequilibrium between the CYP2C8*3 and CYP2C9*2 alleles, forming a haplotype of ~300 kilobases. This haplotype is associated with altered metabolism of several drugs, most notably reduced metabolism of warfarin and phenytoin, leading to toxicity at otherwise therapeutic doses. Here we show that this haplotype is inherited from Neandertals.

## Introduction

The admixture between Neandertals and modern humans that took place ~60,000 years ago introduced genetic variants into the gene pool of modern humans, many of which are still found at low frequencies among present-day people [1]. Prior to what is now estimated to be several admixture events between Neandertals and modern humans, these two groups evolved largely independently of each other for ~500,000 years [2]. During this time, both groups accumulated genetic variants that differed from the ancestral states seen in other primates. Whereas modern humans evolved on the African continent, Neandertals evolved in Eurasia. The different habitats of these two groups might have exerted different evolutionary pressures, leading to the fixation of genetic variants advantageous in the respective environments. Other variants, even slightly deleterious variants, reached fixation due to genetic drift, particularly in periods when population sizes were small. Genetic evidence suggests that the effective population size of Neandertals was considerably smaller than that of modern humans [3].

The *CYP2C9* gene, which encodes the cytochrome P450 enzyme CYP2C9, is highly polymorphic in present-day humans. More than 20 single nucleotide polymorphisms (SNPs) affecting the enzymatic activity of CYP2C9 have been reported. Importantly, people with lower enzymatic activity are at risk of toxic reactions from standard doses of warfarin and phenytoin, which are substrates of the enzyme [4]. The most frequent *CYP2C9* allele, CYP2C9*1, is present at a frequency of 88 % in European populations [5]. The variant CYP2C9*2 with a cysteine replacing an arginine at position 144 in the encoded protein (R144C) at a frequency of 12% in Europe [5]. Its enzyme activity is reduced by ~70% relative to the common CYP2C9*1 allele [6]. Thus, carriers of CYP2C9*2, particularly if homozygous, have been denoted “slow metabolizers” [6].

Approximately 50 kilobases upstream of the *CYP2C9*, is the gene *CYP2C8*, which encodes the cytochrome CYP2C8. This enzyme is a crucial part of the metabolism of several pharmacological agents [7] including antidiabetic drugs (e.g., pioglitazone), statins (e.g., cerivastatin), anti-inflammatory drugs (e.g., ibuprofen) and chemotherapeutic agents (e.g., paclitaxel). The most studied allele in *CYP2C8* is CYP2C8*3, which is characterized by the replacement of an arginine by a lysine at position 139 and a lysine by an arginine at position 399 (R139K and K399R) in the encoded protein. The effect of these variants are substrate dependent, with increased metabolism of drugs such as pioglitazone but decreased metabolism of R-ibuprofen [7]. The two variants in CYP2C8, as well as the R144C variant in CYP2C9, differ not only from the most common alleles in humans but also from the alleles present in apes and humans indicating that they are “new”, i.e., “derived”, changes that occurred recently in evolutionary terms.

The two variant alleles CYP2C9*2 and CYP2C8*3 have previously been shown to frequently co-segregate in families [8]. However, the distance between the R144C variant (chr10:96,702,047_C>T, *hg19*), defining CYP2C9*2, and the variant K399R (chr10:96,798,749_T>C, *hg19*), one of the two variants defining the CYP2C8*3 allele, is 96.7 kilobases. Thus, if these variants co-segregate they are present on an unusually long haplotype. Some such long haplotypes have been introduced by gene flow from Neandertals [1]. Here we verify the co-inheritance of these two alleles in large databases and test the hypothesis that this long haplotype is inherited from Neandertals.

## Methods

Linkage disequilibrium (*r*^2^), i.e., the co-segregation of the alleles, was assessed using the phase 3 release of the 1000 Genomes Project [9], comprising 2504 individuals of different ancestries. Gene-flow from Neandertals was inferred using previously described methods and parameters [10]. In brief, genomic sequences were compared to the corresponding sequences of all available high-coverage Neandertal genomes and the alternative explanation of incomplete lineage sorting was tested using the recombination rate in the genomic region and the length of the haplotype. Allele frequencies were estimated using the 1000 Genomes Project. The four high-coverage Neandertal and Denisovan genomes available were used to assess sequence similarity to present-day haplotypes [11–14]. Single nucleotide polymorphisms at 278 sites in the 1000 Genome project for which there were homozygous variant calls in the Vindija Neandertal in the genomic region chr10:96,537,863–96,851,277 (*hg19*) were used to estimate a phylogeny using a distance-based approach (FastME, version 2.0) and the substitution model of Tamura-Nei [15]. The phylogeny was rooted in the ancestral sequence taken from Ensembl [16].

## Results

When examining the allele frequencies of CYP2C9*2 and CYP2C8*3 across the 1000 Genomes Project we find that both alleles are found in European, Asian and ad-mixed American populations but not in the sub-Saharan population (Table 1). The highest allele frequency (12%) is seen in Europeans in agreement with previous data [5]. We also observe that the frequencies of the two alleles are identical for each population, suggesting co-segregation. Indeed, we find that the two amino acid replacements defining CYP2C8*3 are in perfect linkage disequilibrium (*r*^2^ = 1.0, *p* < 0.001). These two variants are in turn highly correlated with the CYP2C9*2 allele (*r*^2^ = 0.85, *p* < 0.001), defined by the amino acid replacement variant in *CYP2C9*.

**Table 1:** Allele frequencies (A) and linkage disequilibrium (B) between CYP2C8*3 and CYP2C9*2.

A	Variant	EUR	SAS	EAS	AMR	AFR
CYP2C8*3	R139K (rs11572080)	0.12	0.03	0	0.1	0.01
CYP2C8*3	K399R (rs10509681)	0.12	0.03	0	0.1	0.01
CYP2C9*2	R144C (rs1799853)	0.12	0.03	0	0.1	0.01
**B**	**Variant**	**rs11572080**	**rs10509681**	**rs1799853**		
CYP2C8*3	R139K (rs11572080)	1	1	0.85		
CYP2C8*3	K399R (rs10509681)	1	1	0.85		
CYP2C9*2	R144C (rs1799853)	0.85	0.85	1		

Next, we measured the length of the haplotype by finding the set of alleles, which are in linkage disequilibrium (*r*^2^ > 0.8) in the 1000 Genomes individuals. We find that the haplotype spans 313,414 base pairs (chr10:96,537,863-96,851,277 *hg19*). A long haplotype found only among people with genetic roots outside sub-Saharan Africa may be a result of gene flow from Neandertals. We thus examined if the variants of CYP2C9*2 and CYP2C8*3 were present in Neandertal genomes. We find that all three Neandertal genomes sequenced to high coverage carry these variants homozygously at the three positions, except for the 120,000-year-old Neandertal from Denisova Cave in the Altai mountains which is heterozygous for CYP2C9*2 [14]. In contrast, the one genome available from the Denisovans, the Asian sister group of Neandertals [17], harbors the common alleles in homozygous form for two of the three positions, but is homozygous for the derived allele causing R139K in CYP2C8.

**Fig. 1:**
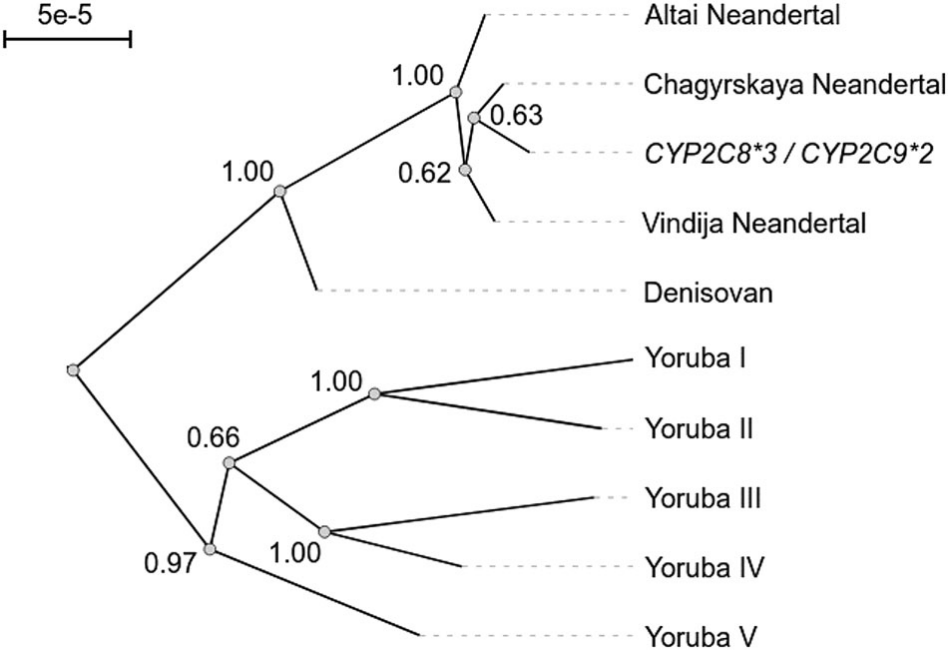
Phylogeny of sequences spanning the genomic region encoding CYP2C8*3 and CYP2C9*2. The tree contains five haplotypes from five present-day day Yoruban (a Nigerian population with minimal Neandertal gene-flow) individuals, one haplotype from an individual of European ancestry carrying CYP2C8*3 and CYP2C9*2, and four sequences from Neandertals and their sister group Denisovans. The sequence with the CYP2C8*3 and CYP2C9*2 alleles is most similar to the corresponding sequence of a Neandertal from the Chagyrskaya Cave in Siberia [11]. Numbers indicate bootstrap support (1000 replicates). Scale bar shows mutations per site. The haplotypes span the genomic region chr10:96,537,863–96,851,277 (*hg19*).

Using data from 1257 meiotic events across 146 families [18], we find that the local recombination rate in this region is 0.27 centimorgan per megabase. Given the length of the haplotype and the recombination rate, we conclude that such a long haplotype is unlikely to have survived recombination since the common ancestor of modern humans and Neandertals (*p* = 1.5e-3). If this haplotype has been introduced to modern humans by gene flow from Neandertals, it should be more closely related to Neandertal haplotypes than to other present-day haplotypes. To clarify this, we estimated the phylogeny for sequences spanning the region chr10:96,537,863-96,851,277 for one European haplotype carrying CYP2C9*2 and CYP2C8*3, five Yoruban haplotypes, one Denisovan haplotype and three Neandertal haplotypes (Fig. 1). The haplotype carrying CYP2C9*2 and CYP2C8*3 share a common ancestor with the Neandertals to the exclusion of the other present-day haplotypes (bootstrap support 1000/1000). Thus, the CYP2C9*2 and CYP2C8*3 alleles were frequent in Neandertals and were later introduced into modern humans when these two groups met.

## Discussion

Over the decade since the first Neandertal genome was sequenced [1], gene flow from Neandertals has been shown to influence many traits, such as skin pigmentation and neurological and psychiatric phenotypes (e.g., [19]). However, the clinical impact of Neandertal genetic variants is less well elucidated, especially in terms of genetic variants with strong effect sizes that need to be taken into account in clinical practice. Recently, the major genetic risk factor for a severe outcome of COVID-19 infection as well as a protective variant have been shown to be of Neandertal origin ([10, 20]). Here we show that two of the most important alleles in pharmacogenetics are inherited from Neandertals. Although this knowledge itself does not change clinical practice, it explains differences observed across ancestries seen in clinical practice.

## Data Availability

The modern human genomes (i.e., the 1000 genomes project) are available at https://www.internationalgenome.org/ and the archaic genomes are available at http://cdna.eva.mpg.de/neandertal/. The recombination rate data can be found at https://www.decode.com/addendum/.
